# Multidimensional LDCT Imaging Endpoints for a Randomized Pilot Phase II Trial of Curcumin and Omega-3 Fatty Acids for Lung Cancer Chemoprevention: Results of a Randomized Pilot Trial

**DOI:** 10.3390/cancers18162565

**Published:** 2026-08-10

**Authors:** Nagi B. Kumar, Matthew Schabath, Mark Alexandrow, Jhanelle Gray, Tawee Tanventyanon, Farah Khalil, José Laborde, Michael J. Schell, Donald Klippenstein

**Affiliations:** 1 Cancer Epidemiology Program, Moffitt Cancer Center and Research Institute, Tampa, FL 33612, USA; matthew.schabath@moffitt.org; 2 Genitourinary Oncology Program, Moffitt Cancer Center and Research Institute, Tampa, FL 33612, USA; 3 Molecular Biology Program, Moffitt Cancer Center and Research Institute, Tampa, FL 33612, USA; mark.alexandrow@moffitt.org; 4 Thoracic Oncology Program, Moffitt Cancer Center and Research Institute, Tampa, FL 33612, USA; jhanelle.gray@moffitt.org (J.G.); tawee.tanventyanon@moffitt.org (T.T.); 5 Pathology, Moffitt Cancer Center and Research Institute, Tampa, FL 33612, USA; farah.khalil@moffitt.org; 6 Biostatistics and Bioinformatics Program, Moffitt Cancer Center and Research Institute, Tampa, FL 33612, USA; jose.laborde@moffitt.org (J.L.); michael.schell@moffitt.org (M.J.S.); 7 Thoracic Radiology, Moffitt Cancer Center and Research Institute, Tampa, FL 33612, USA; donald.klippenstein@moffitt.org

**Keywords:** lung cancer chemoprevention, low-dose CT, lung nodules, curcumin, ω-3 fatty acids, smokers

## Abstract

**Background:** Former and current smokers in lung cancer screening remain at elevated risk for lung cancer despite smoking cessation. We and others have shown that curcumin (CUR) exhibits anti-inflammatory and antiproliferative effects but is limited by poor bioavailability. However, since CUR is lipophilic, co-administration with ω-3 FAs represents a mechanistically rational strategy to enhance delivery and target complementary pathways, including signal transducer and activator of transcription 3 (STAT3) and the transcription factor NF-κB (NF-κB) signaling for lung cancer chemoprevention. **Methods:** We conducted a randomized, single-blind, placebo-controlled Phase II pilot study evaluating CUR combined with ω-3 FAs in high-risk former and current smokers with CT-detected pulmonary nodules. Participants received intervention agents with active-dose groups (low dose = 3; high dose = 9) or a placebo (n = 7) for 6 months. Primary endpoints included radiologic changes in nodule size, number, and density. Secondary endpoints included safety, adherence to the study agent and exploratory biomarker analyses. Correlation analyses of imaging-derived metrics were performed to assess relationships among LDCT parameters. **Results:** Nineteen participants were enrolled (intervention, n = 12; placebo, n = 7). Eighteen (11 intervention, 7 placebo) subjects completed post-intervention imaging. One subject was unable to complete follow-up and study-related procedures. Data from the treatment arms were pooled for analysis and comparison with the placebo arm. No statistically significant between-group differences were observed in primary imaging endpoints. The intervention was well tolerated, with predominantly grade 1 adverse events. Exploratory analyses demonstrated consistent positive correlations among established imaging biomarkers, with clustering of size-based metrics (mean diameter, volume, sum of longest diameters) and density-based parameters. Multidimensional scaling supported this structure, indicating internal coherence among imaging-derived endpoints. **Conclusions:** Although no statistically significant treatment effect on the image biomarkers was observed, this pilot study demonstrates feasibility challenges and identifies coherent imaging biomarkers that may serve as intermediate endpoints in early-phase chemoprevention trials. These results support further investigation of strategies utilizing agent combinations with enhanced bioavailability and safety and refinement of trial design in high-risk lung cancer patient populations.

## Simple Summary

Former and current smokers undergoing lung cancer screening remain at elevated risk despite smoking cessation. Curcumin (CUR) has anti-inflammatory and antiproliferative properties but limited bioavailability. Because CUR is lipophilic, co-administration with omega-3 fatty acids (ω-3 FAs) may enhance absorption while targeting complementary pathways, including NF-κB and STAT3 signaling. We conducted a randomized, single-blind, placebo-controlled pilot trial in high-risk current and former smokers with computed tomography (CT)-detected pulmonary nodules. Participants received CUR plus ω-3 FAs or a placebo for 6 months. Primary endpoints were changes in nodule size, number, and density by low-dose CT. Secondary endpoints included safety, adherence, bioavailability, and exploratory biomarker analyses. Nineteen participants were enrolled (intervention, n = 12; placebo, n = 7), with one subject unable to complete follow-up and study-related procedures. No significant differences were observed in imaging endpoints. Treatment was well tolerated with predominantly grade 1 adverse events. Imaging metrics demonstrated strong correlations among size- and density-based measures, supported by multidimensional scaling analyses. Although efficacy was not demonstrated, this pilot establishes feasibility, confirms the safety of the combination, and identifies coherent imaging biomarkers that may serve as intermediate endpoints for future lung cancer chemoprevention trials.

## 1. Introduction

### 1.1. Current Chemoprevention Options for Former and Current Smokers

Lung cancer remains the leading cause of cancer-related mortality among men and women in Florida, the United States, and worldwide [1,2]. Current strategies to reduce mortality in high-risk populations include lung cancer screening via low-dose computed tomography (LDCT) screening and tobacco control interventions. Approximately 80–90% of lung cancers occur in individuals with a history of smoking, and over 50% arise in former smokers. Tobacco smoking is the most important and prevalent lung cancer risk factor. However, former and current smokers, particularly those undergoing active surveillance for pulmonary nodules, remain at persistently elevated risk and are highly motivated to pursue additional risk-reduction strategies [3].

Chemoprevention during this window represents a rational strategy to target early carcinogenic pathways while evaluating longitudinal changes in imaging-defined pulmonary nodules as candidate intermediate imaging endpoints, thereby complementing LDCT screening and smoking cessation. The rationale for chemoprevention in current and former smokers is supported by several interrelated concepts: (a) lung carcinogenesis arises within airway epithelial cells exposed to shared genetic and environmental insults; (b) the “field cancerization” paradigm suggests that the entire airway epithelium is at risk and amenable to systemic intervention; (c) histopathologic imaging defined abnormalities such as metaplasia and dysplasia can be reproducibly identified and graded [1,4,5]; (d) resolution of smoking-induced inflammation is an active, regulated process mediated by specialized pro-resolving lipid mediators (SPMs) [6,7]; (e) progression to malignancy requires sustained proliferation and evasion of apoptosis driven by inflammatory and growth signaling pathways; and (f) interventions in disease-free, high-risk individuals must promote repair without immunosuppression and with minimal toxicity [2,7,8].

Based on these principles, phytochemicals and biologically active agents capable of modulating inflammatory and proliferative pathways have emerged as promising candidates for lung cancer chemoprevention.

### 1.2. Curcuminoids in Lung Cancer Chemoprevention

Curcumin (diferuloylmethane) (CUR), the active component of *Curcuma longa*, modulates multiple signaling pathways implicated in carcinogenesis, including cell cycle regulation, apoptosis, proliferation, survival, angiogenesis, invasion, and inflammation [9,10,11,12,13,14,15,16,17,18,19]. In particular, signal transducer and activator of transcription 3 (STAT3), activated in approximately 50% of lung cancers, plays a central role in inflammation-driven tumorigenesis [20,21,22,23]. Our preclinical studies demonstrate that CUR suppresses STAT3 phosphorylation and reduces proliferative markers [cyclin D1, Mini-chromosome Maintenance Complex Component 2 (MCM2)] in lung cancer cell lines and murine models in a dose-dependent manner [16]. CUR also inhibits NF-κB signaling through suppression of IκB-α kinase activity and downregulation of pro-inflammatory cytokines [Tumor Necrosis Factor alpha (TNF-α), Interleukin-1, -6 and -8 (IL-1, IL-6, IL-8)] and enzymes (Cyclooxygenase-2 (COX-2), 5-Lipoxygenase (5-LOX)) [10,12,19,24,25,26,27,28,29]. In lung cancer cell lines with constitutive STAT3 and NF-κB activation, CUR effectively reduces inflammatory signaling and proliferation [30].

Preclinical models further demonstrate that CUR suppresses KRAS-driven lung tumor progression and inflammation, supporting its role in delaying malignant transformation [31,32]. CUR has also shown antioxidant and antifibrotic effects in lung tissue without impairing tumor cell killing [3]. Clinical studies confirm favorable safety and bioavailability profiles [33,34,35]. Phase II data demonstrate biological activity, including a 40% reduction in aberrant crypt foci with CUR administration [36]. Novel formulations combining CUR with ω-3 FAs improve systemic and pulmonary bioavailability and reduce tumor growth in murine lung cancer models [37]. Concurrent advances in epigenetics have further demonstrated that DNA methylation, histone modification, chromatin remodeling, and non-coding RNA networks collectively govern cancer-cell plasticity and stemness [38]. Within this contemporary framework, curcumin-mediated inhibition of STAT3/NF-κB signaling may influence not only cellular proliferation but also the epigenetic and transcriptional programs that sustain stem-like phenotypes and adaptive cellular reprogramming, thereby providing a broader biological rationale for cancer interception strategies [39,40].

### 1.3. Omega-3 Fatty Acids in Lung Cancer Chemoprevention

Omega-3 fatty acids (ω-3 FAs) exert anti-inflammatory and pro-resolving effects central to lung carcinogenesis [6,7,41]. A key mechanism involves inhibition of NF-κB signaling, a regulator of inflammatory cytokines, adhesion molecules, and COX-2 expression [42,43,44,45,46]. ω-3 FAs reduce NF-κB activation by preventing IκB phosphorylation and nuclear translocation, thereby decreasing TNF-α and other pro-inflammatory mediators [43,44]. In clinical studies, ω-3 FA supplementation attenuates NF-κB activation, reduces proteasome activity, and lowers inflammatory cytokine levels in cancer patients [43]. Additional studies demonstrate reduced cytokine release and inflammatory signaling in human alveolar cells [45,46,47]. Importantly, ω-3 FAs serve as precursors to specialized pro-resolving mediators (SPMs), including resolvins, protectins, and maresins, which actively promote resolution of inflammation and tissue repair without immunosuppression [6,7,48,49,50]. Because ω-3 fatty acids serve as precursors for specialized pro-resolving mediators (SPMs), clinical supplementation increases circulating levels of Resolvin E1, Resolvin D1, and Resolvin D2 (RvE1, RvD1, and RvD2) [51,52,53], supporting the biological plausibility of enhancing endogenous inflammation-resolution pathways. These mechanisms provided a strong rationale for targeting persistent inflammation in former and current smokers.

### 1.4. Mechanistically Complementary Effects of Curcumin and Omega-3 Fatty Acids

The central limitation of CUR is its poor systemic and tissue bioavailability, which has hindered its translation from promising preclinical findings to consistent clinical efficacy. One of the most compelling strategies to overcome this limitation is co-administration with lipids, particularly ω-3 FAs [28,54]. CUR is inherently lipophilic, and its absorption is markedly enhanced in lipid-rich environments, providing a strong pharmacokinetic rationale for the combination with ω-3 FA. Co-administration facilitates improved solubilization and intestinal absorption, leading to enhanced systemic distribution and more effective delivery to target tissues, including the lungs. Consistent with this, lipid-based formulations combining CUR with ω-3 FAs have demonstrated measurable lung bioavailability and significant reductions in tumor growth in preclinical models [37]. Beyond pharmacokinetic advantages, the combination is supported by complementary and potentially mechanistically complementary biological mechanisms. Both agents modulate key inflammatory pathways, including inhibition of NF-κB signaling, while CUR additionally targets STAT3, and ω-3 FAs contribute to the generation of specialized pro-resolving mediators that actively promote resolution of inflammation [37,54,55,56,57,58]. Preclinical studies demonstrate that co-administration results in greater suppression of cell proliferation, enhanced induction of apoptosis, including increased caspase-3 activity, and more pronounced attenuation of inflammatory signaling compared with either agent alone [37,54,55,56,57,58]. In lung cancer cell models, combined treatment with CUR and ω-3 FAs significantly reduces cell viability relative to single-agent exposure [37]. Importantly, this strategy addresses a key translational barrier while simultaneously amplifying biological efficacy, and both agents have demonstrated favorable safety profiles in clinical settings, supporting the feasibility of this combination as a chemopreventive approach in high-risk populations. To further explore the potential role of the combination of standardized agents—CUR + ω-3 FAs—for lung cancer chemoprevention, we conducted a pilot, randomized, single-blind, placebo-controlled trial in former and current smokers.

### 1.5. Hypothesis and Clinical Translation

Based on this rationale, we evaluated whether a combination of standardized curcumin (CUR) and ω-3 fatty acids (ω-3 FAs) could simultaneously target the STAT3 and NF-κB signaling pathways relevant to lung carcinogenesis. We hypothesized that this mechanistically complementary combination would provide a safe and potentially effective chemopreventive strategy, as assessed by longitudinal changes in pulmonary nodule size, number, and density in current and former smokers. This Phase II pilot trial was designed to evaluate the safety, feasibility, and preliminary biological activity of a standardized combination of curcumin (CUR) and ω-3 fatty acids (ω-3 FAs) for lung cancer chemoprevention in high-risk former and current smokers. Because recruitment ended prematurely when study funding concluded, the low- and high-dose intervention groups were pooled for analysis. Consequently, the trial was not powered to evaluate dose–response relationships or identify an optimal dose. Instead, this study provides preliminary evidence regarding the safety, tolerability, and potential biological activity of the combined intervention and establishes a foundation for adequately powered future randomized trials.

## 2. Materials and Methods

**Figure 1:**
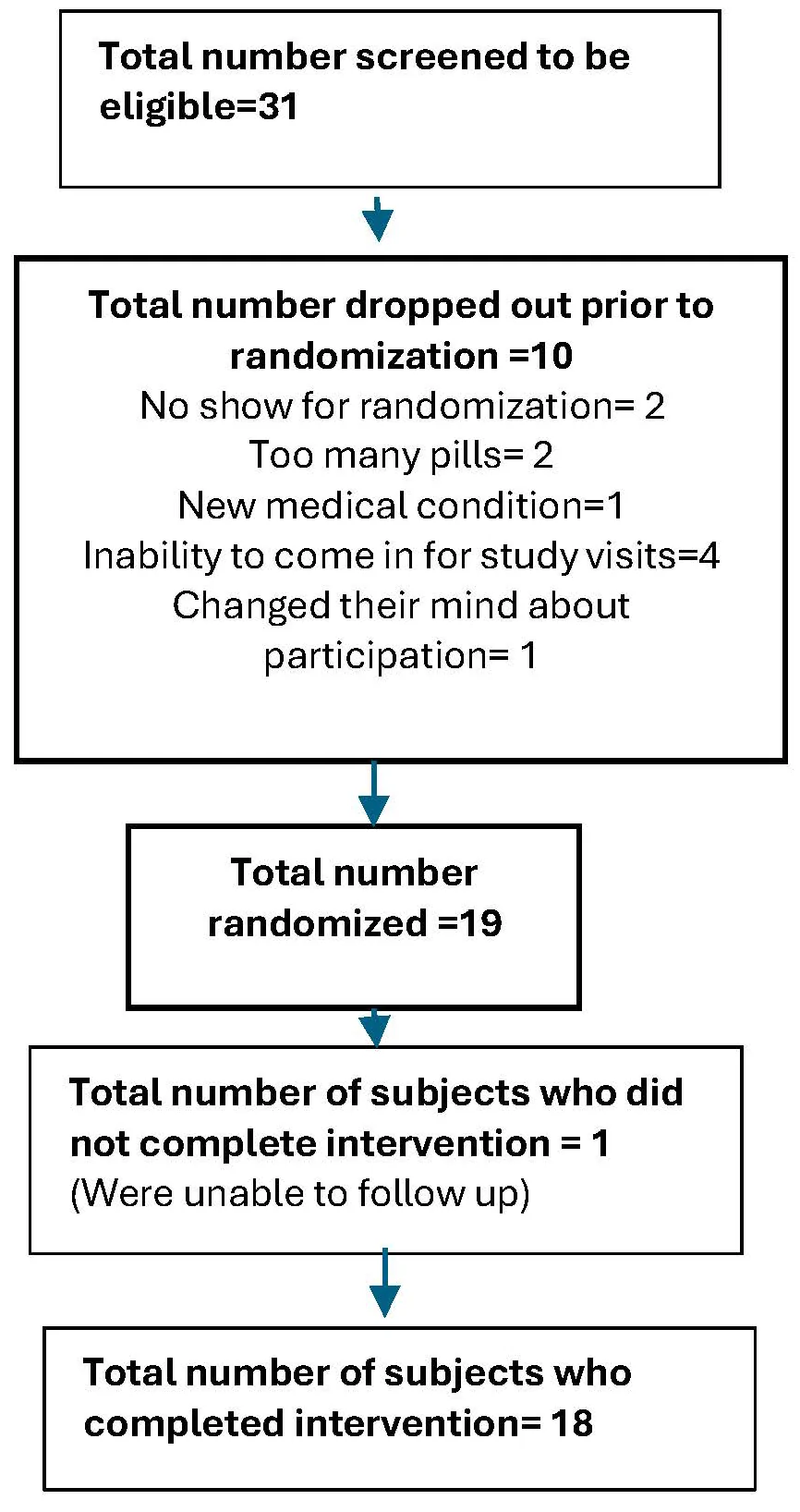
Consort diagram.

Prior to developing the methods for this trial, we consulted with the Patient Advisory Committee for the Thoracic Oncology Program. Based on their recommendation, we conducted a feasibility study to evaluate the interest of this target subject population in participating in an intervention trial [3]. The clinical trial was registered on https://clinicaltrials.gov (trial registration number: NCT03598309; date of registration: 18 July 2018). This study and its consent procedures were approved by the Institutional Review Board, Advara (Approval Code: Pro00028018), on 3 August 2018. A consort diagram depicting the number of subjects screened, enrolled, and randomized and those who completed the intervention is shown in Figure 1. Due to delays because of the COVID epidemic, participants were enrolled at the Moffitt Cancer Center, a National Cancer Institute-designated Comprehensive Cancer Center, from June 2019 to November 2024. Potential participants were identified by the Thoracic Oncology Program and were scheduled for an LDCT in the lung cancer screening program or were being evaluated for a suspicious pulmonary nodule. Criteria for inclusion included male or female, 55 years of age or older who were former or current smokers and enrolled in the lung cancer screening program or those who were diagnosed using a diagnostic CT, and have Lung Imaging Reporting and Data System (Lung-RADS) [59]-based RAD 3 category lesion(s) that would receive a 6-month follow-up LDCT or diagnostic CT based on Lung-RADS recommendations or have Lung-RADS 2 category lesions with lung nodules with ≥4 mm mean diameter detected during screening LDCT or regular CT scan.

Other criteria included a history of cigarette smoking of ≥20 pack-years (equivalent to smoking one pack per day for 20 years, or an equivalent cumulative exposure); Eastern Cooperative Oncology Group (ECOG) performance score of ≤1; able to swallow study agents; willing and able to undergo CT scans; not allergic to components of the study agents; and willing to comply with the requirement to not consume additional dietary ω-3 fatty acids or CUR supplements during the intervention period.

All current smokers were offered smoking cessation services, whether or not they agreed to participate in the trial. Participants were required to have normal organ and marrow function based on screening CBC and CMP. Exclusion criteria included history of lung cancer or other invasive malignancy (with the exclusion of basal cell carcinoma or skin squamous cell carcinoma) and recent use of doxycycline or tetracycline (within ≤2 weeks).

The PI obtained approval from the United States Food and Drug Administration Investigator Initiated New Drug (US FDA IND) (139704) for the combination of study agents. This study was conducted in compliance with the FDA regulatory requirements.

Throughout the trial, we ensured that the criteria for inclusion/exclusion and safety and symptom monitoring procedures ensured the safety of the agent administered to this target population for the duration of intervention. Thus, benefit versus harm was monitored throughout the trial.

### 2.1. Randomization and Blinding

Upon obtaining informed consent, all subjects were screened for eligibility. All screenings of LDCT or diagnostic CTs were categorized by the study radiologist in a blinded fashion. After potential subjects were identified as having at least one lung nodule that met eligibility criteria by the study radiologist (DK), subjects were assigned to the intervention (one of two doses of CUR and ω-3 FAs) or the placebo arm (1:1:1) using the SRAR system, a web-delivered subject registration and randomization application. All study staff and participants, with the exception of the clinical pharmacist and randomization team, were blinded to the assignments until the completion of the trial.

### 2.2. Study Agents

Curcumin C3 Complex^®^ was obtained from Sabinsa Corporation (East Windsor, NJ, USA). Curcumin C3 Complex^®^, collectively known as curcuminoids, is a standardized preparation (Sabinsa Corporation) derived from the ground rhizomes of *Curcuma longa* L (Turmeric), a herbaceous plant and a member of the ginger family (Zingiberaceae). Curcumin C3 Complex^®^ has also been reviewed by the US FDA under an agency response letter (GRAS Notice No. GRN 000460). We used the Curcumin C3 Complex^®^ tablets from a single batch manufactured by the Sabinsa Corporation. C3 Complex^®^ is >95% curcuminoids, composed of curcumin (75–81%), bisdemethoxy curcumin (2.2–6.5%), and desmethoxy curcumin (15–22%). Each tablet contained 1000 mg of curcuminoids (900 mg of curcumin, 80 mg of desmethoxycurcumin, and 20 mg of bisdesmethoxycurcumin), confirmed by HPLC/MS. Bottles were labeled to comply with study requirements and applicable regulations for investigational drug use. Bottles with the study agent were stored at controlled room temperature (15–30 °C).

We utilized a standardized product, Lovaza^®^ (omega-3-acid ethyl esters), an FDA-approved prescription omega-3 that provides prescription-only potency and purity; each 1 g Lovaza^®^ (omega-3-acid ethyl esters) gel capsule contains 90% omega-3-acid ethyl esters and 84% eicosapentaenoic acid (EPA) and docosahexaenoic acid (DHA) ethyl esters. Lovaza^®^ was provided by GlaxoSmithKline (London, UK), which has authorized the FDA Center for Drug Evaluation and Research, Division of Metabolic and Endocrine Drug Products, to reference Lovaza^®^ Capsules IND # 45,998.

The placebo tablets matching Curcumin C3 Complex^®^ were supplied by Sabinsa Corporation. The placebo used #1 gelatin capsules containing microcrystalline cellulose with the coloring material Yellow Lake L-1200200 (Colorcon). Matching placebo capsules for Lovaza^®^ (#1 gelatin capsules containing microcrystalline cellulose) were prepared by Carrollwood Compounding Pharmacy (Tampa, FL, USA).

Both intervention groups received the same daily dose of Curcumin C3 Complex^®^ (8 g/day administered in two divided doses); the only difference between the intervention groups was the dose of omega-3 fatty acids. Group A received Lovaza^®^ 4 g/day plus Curcumin C3 Complex^®^ 8 g/day, administered in two divided doses. Group B received Lovaza^®^ 2 g/day plus Curcumin C3 Complex^®^ 8 g/day, administered in two divided doses. The placebo group received matching placebos for both Lovaza^®^ and Curcumin C3 Complex^®^, administered in two divided doses.

An investigator-initiated IND (139704; Kumar NB, PI) was obtained for this combination of agents (curcumin plus ω-3 fatty acids) at the doses administered in this trial for this indication (12 July 2018). Annual testing of the study agents confirmed stability and maintenance of full potency through 2025, when the final participant completed the trial. To minimize the use of non-study supplements, all participants received a standard vitamin and mineral formulation providing 100% of the US Recommended Daily Allowance throughout this study.

### 2.3. Intervention

The intervention was administered on an outpatient basis. Intervention agents were self-administered. Instructions regarding the administration of study agents and potential side effects were provided to the subjects by the study team. Subjects were instructed to limit intake of or avoid other supplements containing CUR or ω-3 FAs. We monitored compliance with these instructions throughout the trial using daily agent intake logs and 2-day food records completed each week. Subjects were required to (a) maintain ≥85% compliance with study agent intake, (b) comply with dietary, medication and supplement restrictions, and (c) complete the study forms and daily logs. Incidence of adverse events and toxicities, monitored using Common Toxicity Criteria (CTCAE) version 5.0, and the results of complete blood count (CBC) and comprehensive metabolic panel (CMP) were assessed prior to the start of the trial, at the midpoint (3 months), and at the end of the trial (6 months). Drug administration continued from the time of randomization until the time of LDCT or regular CT at 6 months or earlier if medically indicated; the planned/maximum duration of intervention was 6 months. Medical indications for discontinuing intervention included intolerable toxicity, development of lung cancer or withdrawal due to other reasons not communicated by the subject to the study team. All subjects were contacted 7 ± 3 days following the intervention to assess toxicity and concomitant medications.

### 2.4. Study Endpoints

The primary endpoint was to evaluate the effectiveness of combination agents (Curcumin C3 Complex^®^ + Lovaza^®^ vs. placebo) administered for 6 months to asymptomatic former and current smokers with lung nodules (detected during LDCT or detected using diagnostic CT), as indicated by (a) a change in size of CT-detected lung nodules, (b) the number of nodules with a longest diameter of ≥ 4 mm, and (c) the lung density of solid, partially solid and non-solid nodules between the treatment and placebo arms. Secondary endpoints included safety of the combination agents (Curcumin C3 Complex^®^ + Lovaza^®^ vs. placebo) as indicated by incidence of adverse events and toxicities, monitored using Common Toxicity Criteria (CTCAE) version 5.0 throughout the trial, and the results of complete blood count (CBC) and comprehensive metabolic panel (CMP) at the baseline, the midpoint, and at the end of the trial. Additionally, we included adherence and acceptability of the combination agents (vs. placebo), as indicated by 85% compliance with the study agent and supplement and dietary restrictions. All subjects who were compliant, took 85% of the dose of study medication, and underwent a post-treatment CT were included in the efficacy analysis. All participants included in this study were assessed for response to intervention, even if there were major protocol deviations. All conclusions regarding efficacy were based on all eligible participants.

### 2.5. Measurement of Lung Nodules

Each lung nodule was measured during baseline screening and 6-month LDCT or standard CT with 1 mm thick axial images using standard lung window settings (C-500 HU, W 1500 HU), with the nodule diameter representing the average of the longest nodule diameter and the perpendicular diameter. New lung nodules at the 6-month LDCT or diagnostic CT were measured on the screening CT if they were present, in retrospect. Both the overall nodule size and the size of the solid component of the nodule were measured and recorded for part-solid lung nodules. For both part-solid and non-solid lung nodules, the average density of the solid and non-solid portions of the nodule was determined during screening and 6-month LDCT or standard CT. In addition, all lung nodules ≥ 4 mm during screening and 6-month LDCT or standard CT were assessed for a change in the average diameter of >1 mm. The data were summarized as the average change in the size of nodules (primary endpoint) from the LDCT or standard CT. In addition, we observed trends in the number and size of nodules ≥ 4 mm and the lung nodule density of partially solid and non-solid nodules between the intervention and placebo arms.

### 2.6. Data Management and Study Monitoring

All collected data were entered from source documents or case report forms (CRFs) directly into the web-based ONCORE system by authorized, trained staff. Toxicities were monitored continuously through the trial by the PI and study physicians. This study was monitored in accordance with the Protocol Review and Monitoring System at the Moffitt Cancer Center.

As required by NIH rules, we will make the data collected as a part of this protocol available to outside investigators. In order to maintain compliance with HIPAA regulations, our preferred method will be to execute a data sharing agreement with the requestor for a limited use dataset as defined by the US Department of Health and Human Services (DHHS).

### 2.7. Statistical Methods

The analytic dataset included 19 participants (Group A, n = 3; Group B, n = 9; Group C, n = 7). For analysis, Groups A and B (curcumin + ω-3 fatty acids) were pooled and compared with Group C (placebo). Given the pilot nature of this study and its limited sample size, all analyses were considered exploratory. Baseline demographic and clinical characteristics were summarized using descriptive statistics. Continuous variables are presented as mean (standard deviation) or median (range), as appropriate, and categorical variables as frequencies and percentages.

For imaging endpoints, nodule-level measurements were aggregated at the patient level to account for within-subject correlation. Specifically, baseline-to-end-of-treatment ratios (end-of-treatment divided by baseline) were calculated for each nodule, and summary measures (e.g., sum of longest diameters) were derived at the patient level for analysis.

Between-group comparisons of continuous outcomes were performed using the Wilcoxon rank-sum test (Mann–Whitney U test) due to the small sample size and non-normal distributions. Parametric (Welch *t*-test) and non-parametric (Kruskal–Wallis) tests were additionally performed as sensitivity analyses.

Correlations among LDCT-derived imaging variables were assessed using Spearman rank correlation coefficients, given the non-parametric nature of the data. Correlation matrices were constructed to evaluate pairwise relationships among size- and density-based metrics.

To further explore the structure of relationships among imaging variables, multidimensional scaling (MDS) was applied to the correlation matrix to visualize clustering patterns among related metrics.

Given the hierarchical structure of nodules within patients and the limited sample size, formal mixed-effects modeling was not performed, and analyses were conducted at the patient level. Accordingly, findings should be interpreted as exploratory and hypothesis-generating. All statistical analyses were conducted using R software (version 4.5.0). No adjustments were made for multiple comparisons.

Because of the small sample size, no interim analysis was planned.

## 3. Results

**Participant Characteristics and Study Completion** (Table 1): This study was originally designed as a three-arm pilot trial with planned 1:1:1 randomization to curcumin plus low-dose ω-3 fatty acids (ω-3 FAs), curcumin plus high-dose ω-3 FAs, or a placebo. Recruitment ended when funding for the pilot trial concluded. At study closure, 19 participants were randomized: 3 to the low-dose arm, 9 to the high-dose arm, and 7 to the placebo arm. One subject was unable to complete follow-up and study-related procedures. Because recruitment ended before balanced enrollment across the three treatment groups was achieved, the low-dose arm was too small to permit meaningful dose–response comparisons. Therefore, for the exploratory analyses, the low- and high-dose intervention groups were pooled and compared with the placebo group. No protocol or ClinicalTrials.gov amendment was required because the trial closed for recruitment upon the completion of the funded study period rather than because of a protocol modification.

Baseline demographic and clinical characteristics were generally balanced between groups, with a median age of 69 years (range: 57–75) and a predominance of male participants (57.9%). Study completion was limited by challenges with participant recruitment, adherence, and retention, resulting in a substantial proportion of participants discontinuing before completing this study. These factors resulted in a reduced evaluable sample size for efficacy analyses.

**Safety and Tolerability** (Table 2): The combination of curcumin (CUR) and ω-3 fatty acids was generally well tolerated. A total of seven participants experienced study drug-related adverse events, most of which were grade 1 in severity. Reported events included gastrointestinal symptoms (e.g., flatulence, reflux, and vomiting), fatigue, and mild metabolic abnormalities. No unexpected safety signals or high-grade toxicities attributable to the intervention were observed.

Both intervention groups received the same curcumin dose; the only difference between the groups was the dose of ω-3 fatty acids (low-dose vs. high-dose ω-3 FAs). Accordingly, any apparent differences in adverse event frequencies between the intervention groups should be interpreted with caution, as this study was not designed or powered to evaluate dose-related differences in toxicity, particularly given the small sample size.

**Adherence and Feasibility:** Adherence to the study intervention was variable, as assessed by pill counts and self-reported logs. This study was conducted during the COVID-19 pandemic, which, along with the high comorbidity burden of the study population, contributed to challenges in participant retention and compliance. These factors limited statistical power but provided important feasibility insights relevant to the conduct of chemoprevention trials in high-risk populations.

**Figure 2:**
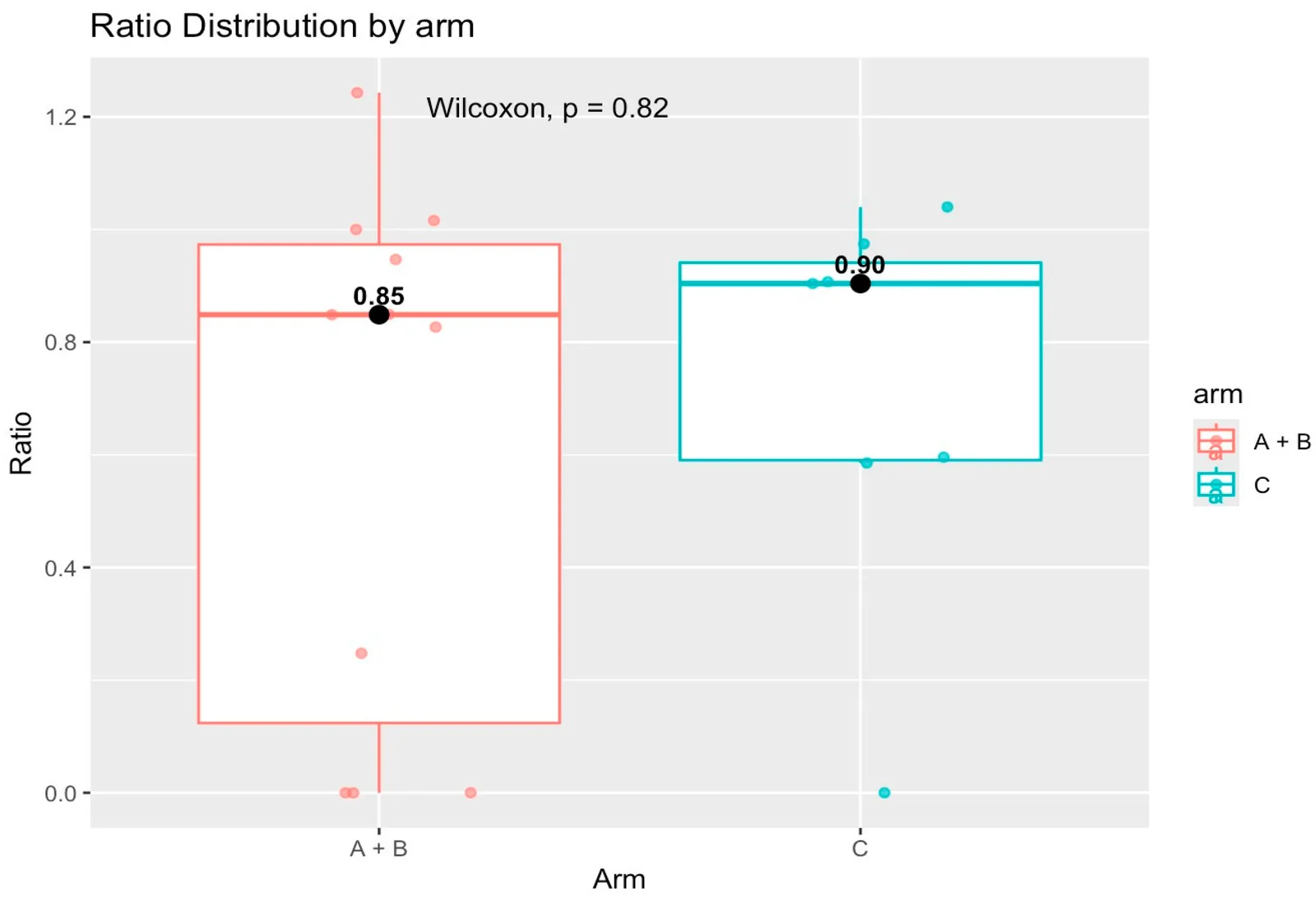
Baseline-to-end-of-treatment ratios of longest diameter for subsolid pulmonary nodules by study arm. Ratios represent end-of-treatment values relative to baseline measurements for each nodule. Distributions are shown separately by study arm. Longest diameter was measured in millimeters on low-dose computed tomography (LDCT).

**Figure 3:**
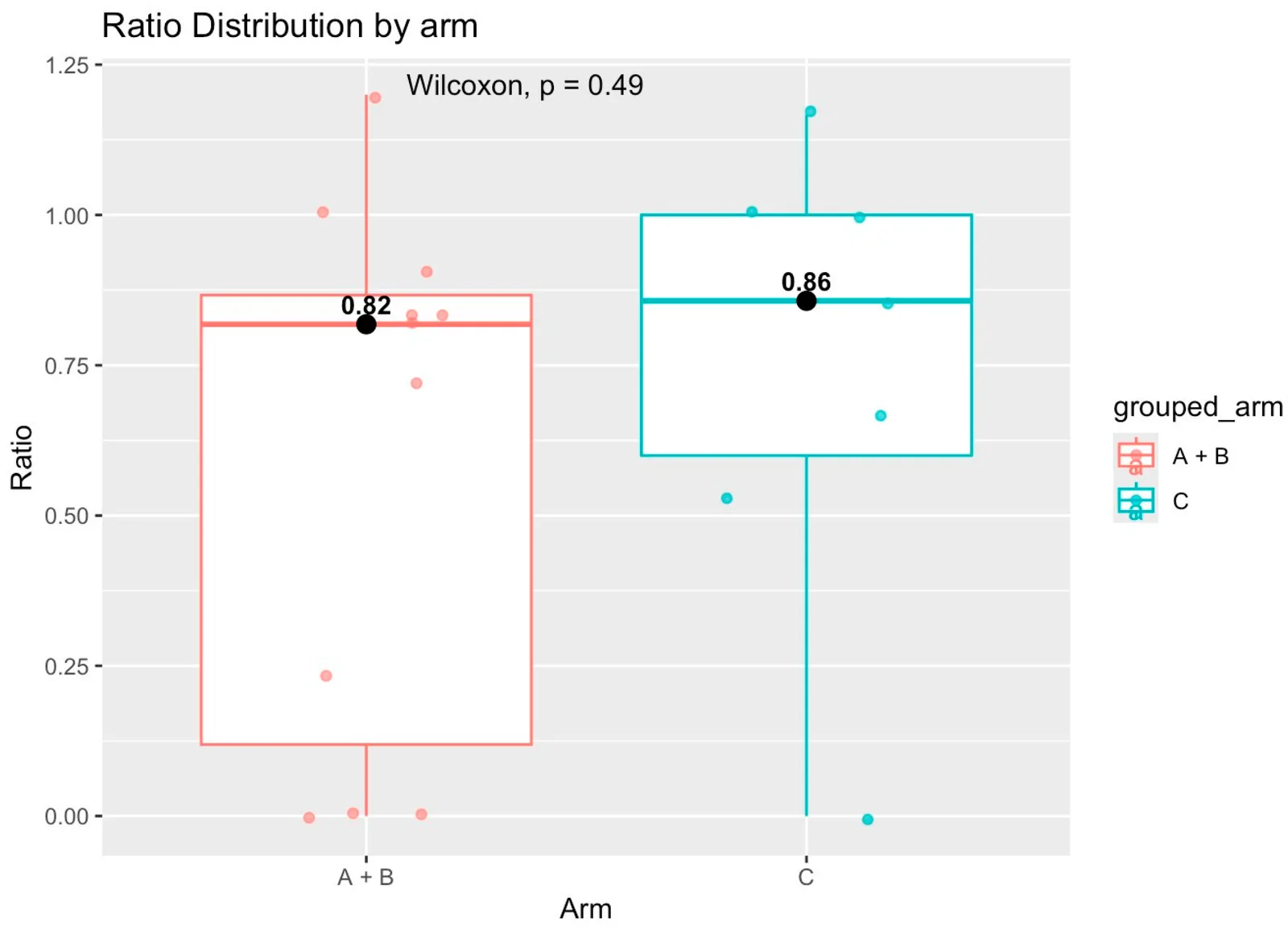
Baseline-to-end-of-treatment ratios of mean nodule diameter by study arm. Mean diameter was calculated for each nodule from orthogonal LDCT measurements. Ratios represent end-of-treatment values normalized to baseline values. Distributions are presented by study arm.

**Figure 4:**
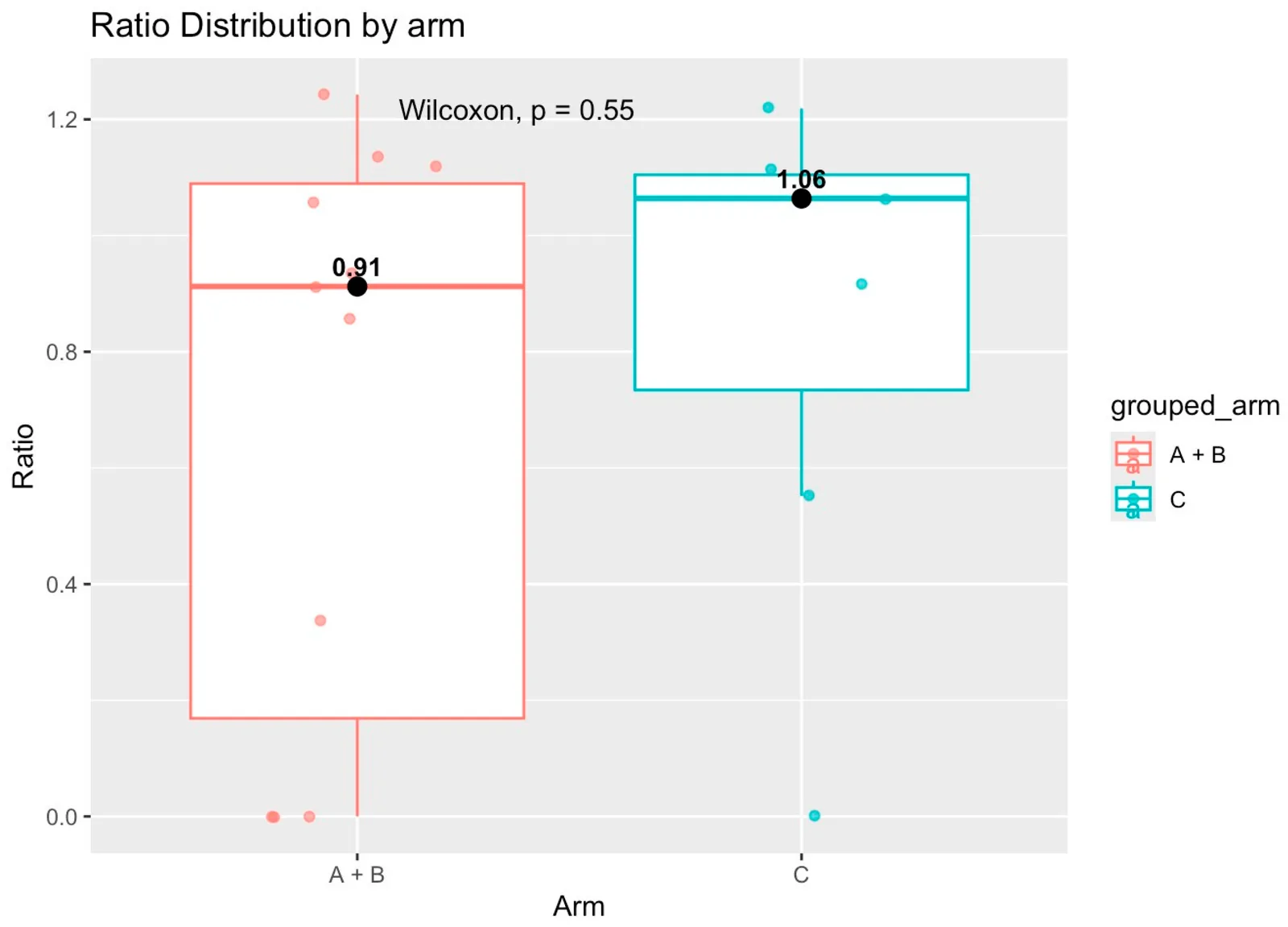
Baseline-to-end-of-treatment ratios of average nodule density by study arm. Average nodule density was measured in Hounsfield units on LDCT. Ratios represent end-of-treatment values relative to baseline measurements. Data are stratified by study arm.

**Primary Imaging Outcomes:** Table 3a provides the overall baseline-to-end-of-treatment ratio of the sum of longest diameters of subsolid nodules. Values are presented as median (range) for all evaluable nodules. Table 3b provides the baseline-to-end-of-treatment ratio of the sum of longest diameters of subsolid nodules by study arm. Values are presented as median (range) by study. Figure 2 presents the baseline-to-end-of-treatment ratios of the longest diameter for subsolid pulmonary nodules by study arm. Ratios represent end-of-treatment values relative to baseline measurements for each nodule. Distributions are shown separately by study arm. The longest diameter was measured in millimeters on low-dose computed tomography (LDCT). Figure 3 presents the baseline-to-end-of-treatment ratios of mean nodule diameter by study arm. Mean diameter was calculated for each nodule from orthogonal LDCT measurements. Ratios represent end-of-treatment values normalized to baseline values. Distributions are presented by study arm. Figure 4 presents the baseline-to-end-of-treatment ratios of average nodule density by study arm. Average nodule density was measured in Hounsfield units on LDCT. Ratios represent end-of-treatment values relative to baseline measurements. Data are stratified by study arm. Table 4 provides the between-group comparisons of baseline-to-end-of-treatment ratios of the sum of longest diameters of subsolid nodules. Values represent patient-level aggregated ratios (end-of-treatment divided by baseline). Between-group comparisons were performed using the Wilcoxon rank-sum test (primary analysis), with Welch *t*-test and Kruskal–Wallis test conducted as sensitivity analyses. *p* values are exploratory and not adjusted for multiple comparisons. Although relatively lower increases in these nodule parameters were observed in the intervention arm compared to the placebo arm, no statistically significant differences were observed between the intervention and placebo groups across primary LDCT-derived imaging endpoints, including changes in nodule size, volume, number of nodules ≥4 mm, and density-related measures. Across all evaluated parameters, distributions of aggregated baseline-to-follow-up ratios were comparable between groups, with no consistent directional trends favoring the intervention.

**Figure 5:**
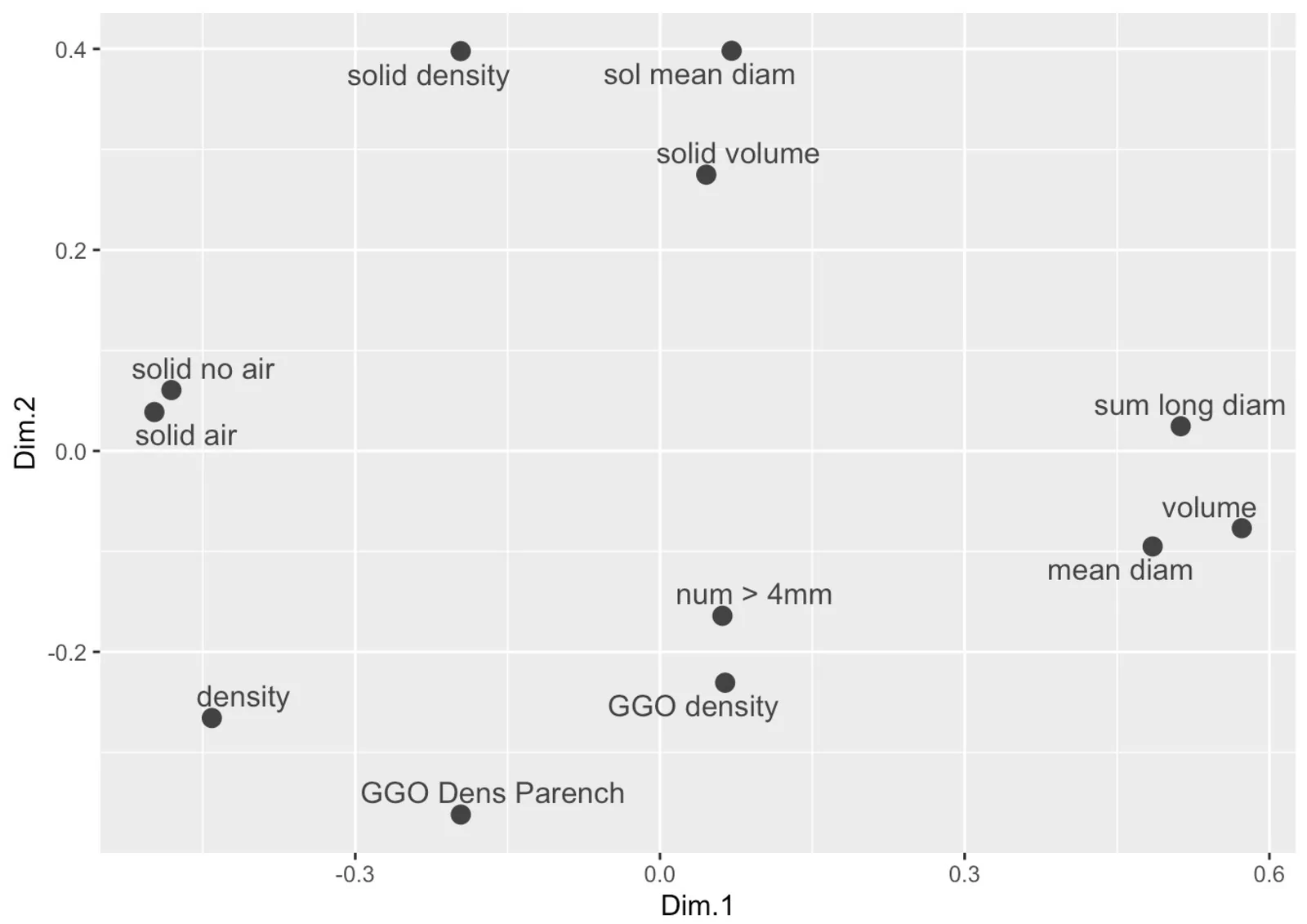
A multidimensional scaling plot illustrating the correlation structure among LDCT-derived nodule characteristics. The multidimensional scaling (MDS) plot was generated from pairwise correlations among LDCT-derived nodule metrics, including the longest diameter, mean diameter, volume, and density-related measures. Variables clustering together demonstrate stronger correlation and shared underlying structure. Distinct clustering of size-related metrics and density-related parameters supports the internal coherence of LDCT-derived imaging endpoints and reinforces their potential utility as complementary imaging biomarkers.

**Figure 6:**
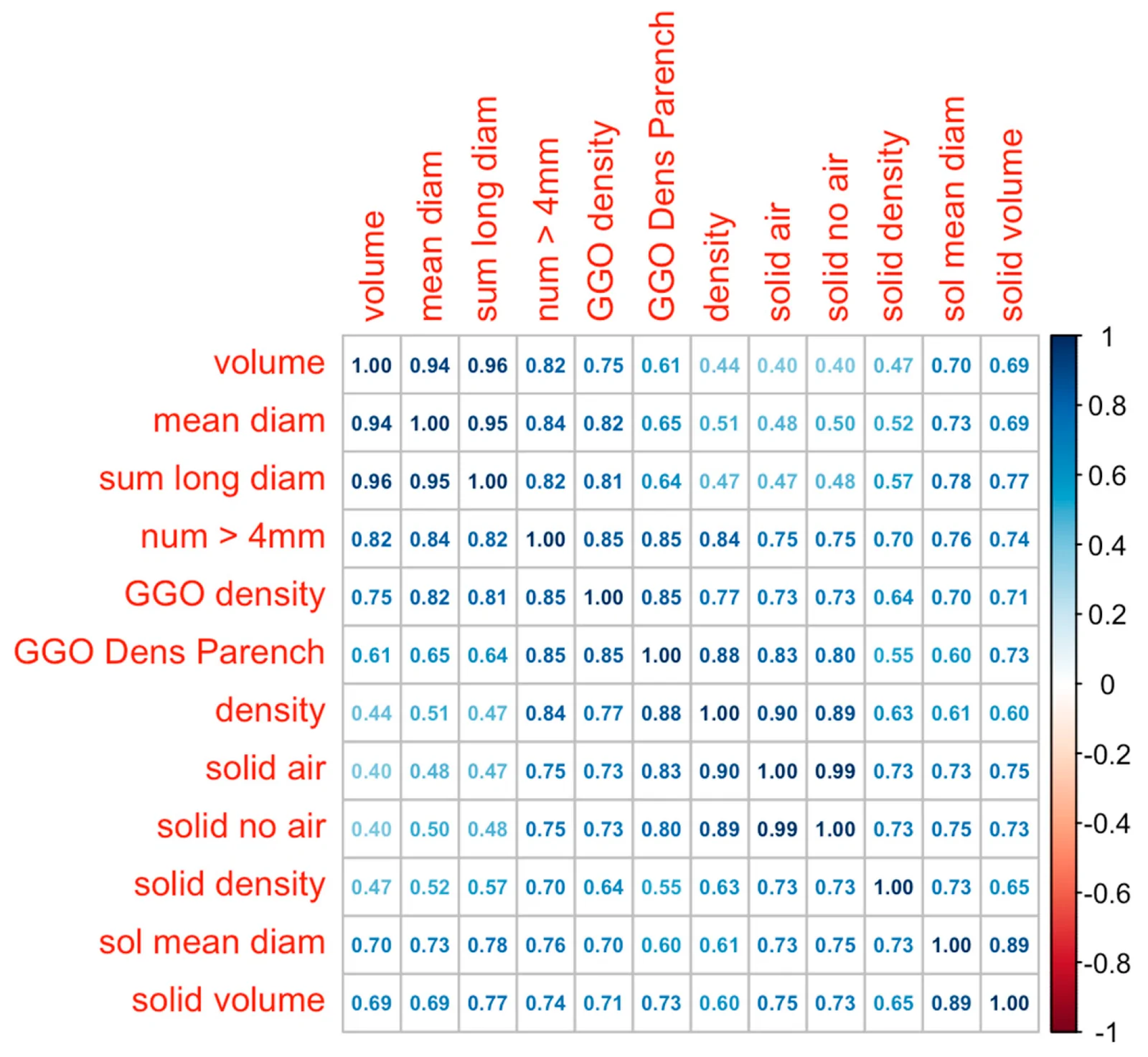
Correlation matrix of LDCT-derived nodule characteristics demonstrating clustering of size- and density-related imaging metrics. Pairwise correlations among LDCT-derived nodule characteristics, including longest diameter, mean diameter, volume, and density-related parameters, are presented. Stronger correlations and clustering among size-related variables and among density-related variables indicate shared underlying structure and internal coherence of LDCT-derived imaging endpoints. Diam = diameter; GGO = ground-glass opacity; Dens = density; Parench = parenchyma.

Exploratory Imaging Biomarker Analyses: Table 5 presents the baseline-to-end-of-treatment ratios of LDCT-derived nodule characteristics by study arm, demonstrating internal consistency across imaging endpoints. Values are presented as median (interquartile range). Ratios are defined as end-of-treatment values relative to baseline measurements. *p* values represent comparisons between study arms. LDCT-derived metrics include size-based parameters (e.g., mean diameter, volume, sum of longest diameters) and density-based parameters (e.g., average density, solid and ground-glass opacity components). Figure 5 displays a multidimensional scaling plot illustrating the correlation structure among LDCT-derived nodule characteristics. The multidimensional scaling (MDS) plot was generated from pairwise correlations among LDCT-derived nodule metrics, including longest diameter, mean diameter, volume, and density-related measures. These exploratory analyses demonstrated consistent positive correlations among LDCT-derived measures of pulmonary nodule characteristics. Strong clustering was observed among size-based metrics, including mean diameter, nodule volume, and the sum of longest diameters, while density-related variables formed a separate but internally consistent cluster. These relationships were visualized through correlation matrix analyses and further supported by multidimensional scaling, which demonstrated spatial grouping of related variables (Figure 6). Collectively, these findings indicate internal coherence among LDCT-derived parameters, suggesting that these measures capture related but distinct dimensions of nodule biology. While not indicative of treatment effect, this consistency supports the potential utility of LDCT-based metrics as candidate intermediate endpoints for signal detection in early-phase chemoprevention studies. These structured interrelationships among LDCT-derived metrics are consistent with prior radiomics studies [60,61] demonstrating clustering of imaging features into biologically relevant domains, supporting the validity of these measures as candidate intermediate endpoints.

## 4. Discussion

In this first pilot randomized study evaluating the combination of CUR and ω-3 FAs in high-risk current and former smokers with imaging-detected pulmonary nodules, we did not observe statistically significant differences between intervention and placebo groups across primary imaging endpoints. However, this study was designed as an exploratory, signal-generating trial, and several findings provide important insights relevant to the design and conduct of future chemoprevention studies.

First, exploratory analyses demonstrated consistent positive correlations among low-dose computed tomography (LDCT)-derived measures of nodule size, volume, and density, suggesting internal coherence among imaging-based endpoints. These relationships, supported by correlation matrix and multidimensional scaling analyses, indicate that LDCT-derived metrics may capture related but distinct aspects of nodule biology and could serve as coherent imaging biomarkers in early-phase chemoprevention trials. Prior radiomics studies have demonstrated that CT-derived imaging features cluster into correlated domains reflecting tumor size, morphology, and density [60,61]. We included patients with Lung-RADS 3 category lesion(s) or those with Lung-RADS 2 category lesions with lung nodules with ≥ 4 mm mean diameter detected during screening LDCT or regular CT scan. These nodules represented imaging-defined abnormalities associated with varying levels of lung cancer risk and were selected as candidate intermediate imaging endpoints for longitudinal assessment. However, they were not histologically confirmed premalignant lesions. Accordingly, changes in nodule size, density, or number cannot be interpreted as direct evidence of regression of premalignant epithelium or reversal of carcinogenesis. Our findings extend these concepts to clinically accessible LDCT-derived metrics, demonstrating that routinely measured parameters exhibit similar internal structures, supporting their use as coherent intermediate endpoints. In settings where clinical endpoints require prolonged follow-up, such imaging-based measures may provide a practical and biologically relevant approach to signal detection and intermediate endpoint evaluation.

Second, despite the absence of a measurable efficacy signal, the biological rationale for the combined use of CUR and ω-3 FAs remains compelling. CUR targets key oncogenic and inflammatory pathways, including STAT3 and NF-κB signaling, while ω-3 FAs modulate inflammatory responses and promote resolution through specialized pro-resolving mediators. Importantly, the lipophilic nature of CUR presents a known limitation to its clinical translation, and its co-administration with ω-3 FAs offers a mechanistically grounded strategy to enhance its bioavailability and tissue delivery. Preclinical evidence supports both improved pharmacokinetics and mechanistically complementary biological activity of this combination, including the enhanced suppression of proliferation and inflammatory signaling. Thus, the absence of observed clinical effects in this pilot study likely reflects limitations in study design and execution rather than a lack of underlying biological activity.

Third, this study highlights critical feasibility challenges inherent to chemoprevention trials in high-risk populations. Conducted during the COVID-19 pandemic, this study experienced substantial barriers pertaining to recruitment, adherence, and retention. The target population—older individuals with a history of smoking and a high burden of comorbidities—represents a clinically relevant but complex group in whom competing health priorities and treatment fatigue may limit sustained participation. These factors contributed to a reduced evaluable sample size and limited statistical power, constraining the ability to detect modest intervention effects. Importantly, these challenges reflect real-world conditions and underscore the need for pragmatic trial designs that incorporate adherence support, simplified intervention strategies, and optimized patient selection criteria.

This study has several limitations. First, as noted above, the sample size was small, and this study was not powered to detect modest between-group differences, consistent with its design as a pilot, exploratory trial. The challenges in recruitment, adherence, and retention—exacerbated by the COVID-19 pandemic and the comorbidity burden of the study population—limited the evaluable sample size and statistical power. Second, pulmonary nodules are clustered within patients; to address this, nodule-level measurements were aggregated at the patient level prior to analysis. Given the limited sample size and number of observations per subject, more complex hierarchical modeling approaches, such as mixed-effects models, were not feasible without risk of overfitting. Third, the primary aim was to evaluate the feasibility and preliminary biological activity of a mechanistically rational intervention using multidimensional LDCT-derived imaging biomarkers as early endpoints of chemopreventive response rather than to optimize combination pharmacokinetics or establish pharmacological synergy. Consequently, pharmacokinetic measurements of CUR or ω-3 FAs, tissue exposure assessments, and formal analyses of drug interactions were not incorporated into the study design. At the time the study was initiated, validated analytical methods for the routine quantification of CUR and its metabolites were also not readily available within our institutional research infrastructure. Accordingly, the present study cannot determine whether co-administration altered CUR bioavailability, prolonged biologically relevant exposure, or achieved pharmacokinetic synchronization between the two agents. These limitations should be addressed in future studies incorporating factorial trial designs, validated LC-MS/MS pharmacokinetic assays, pharmacodynamic biomarkers, and quantitative interaction modeling. Additionally, multiple comparisons were performed without formal adjustment, and findings should therefore be interpreted descriptively. Accordingly, the results should be considered hypothesis-generating and intended to inform the design of future adequately powered studies.

Collectively, these findings emphasize that early-phase chemoprevention trials must balance biological rigor with feasibility. The present study provides preliminary evidence supporting the use of LDCT-derived imaging metrics as potential intermediate endpoints and reinforces the importance of addressing pharmacologic delivery challenges for agents such as CUR. Future studies should build on these insights by employing adequately powered designs, refining intervention delivery approaches to enhance bioavailability, and incorporating strategies to improve adherence in high-risk populations. In addition, further validation of coherent imaging biomarkers in relation to biological and clinical outcomes will be essential to advance their role in chemoprevention research.

## 5. Conclusions

In conclusion, although this pilot proof-of-concept study was not powered to detect statistically significant differences in the primary imaging endpoints, it demonstrates the feasibility of evaluating a mechanistically rational chemoprevention strategy using multidimensional LDCT-derived imaging biomarkers in individuals at increased risk for lung cancer. This study also highlights important methodological considerations for the design of future early-phase lung cancer prevention trials, including the selection of sensitive imaging endpoints, integration of mechanistic biomarkers, and careful evaluation of combination interventions.

These findings support the continued investigation of multidimensional imaging approaches that integrate complementary measures of pulmonary nodule size and density to improve risk stratification and endpoint selection. Future studies should incorporate larger sample sizes, pharmacokinetic and pharmacodynamic assessments, and molecular biomarkers to better define the biological activity of CUR and ω-3 FA-based interventions and to determine their potential role in lung cancer interception.

### Future Directions

The future of cancer chemoprevention depends not only on the identification of effective preventive agents but also on the development and validation of rigorous intermediate endpoints capable of efficiently detecting biological activity in early-phase trials. This pilot study contributes to that objective by demonstrating the feasibility and internal coherence of multidimensional LDCT-derived imaging endpoints while highlighting the practical challenges inherent in conducting prevention studies in high-risk populations. These findings provide a methodological framework for future lung cancer interception trials and support the continued refinement of biomarker-driven trial design.

## Figures and Tables

**Table 1:** Baseline demographic characteristics by study arm. Values are presented as mean (SD), median (range), or number (percentage), as appropriate. *p* values compare differences between study arms.

Variables	Overall (N = 19)	A + B (n = 12) (Intervention Arm)	C (n = 7) (Placebo)	*p*
**Age (years)**				0.075
n	19	12	7	
Mean (SD)	68.5 (5.28)	70.1 (5.18)	65.7 (4.54)	
Median (range)	69.0 (57.0, 75.0)	69.5 (57.0, 75.0)	65.0 (59.0, 73.0)	
**Biological Gender, n (%)**				0.377
Female	8 (42.1%)	4 (33.3%)	4 (57.1%)	
Male	11 (57.9%)	8 (66.7%)	3 (42.9%)	
**Race, n (%)**				0.674
Asian	1 (5.3%)	0 (0.0%)	1 (14.3%)	
Black or African American	1 (5.3%)	1 (8.3%)	0 (0.0%)	
White	15 (78.9%)	10 (83.3%)	5 (71.4%)	
Unknown	2 (10.5%)	1 (8.3%)	1 (14.3%)	
**Ethnicity, n (%)**				>0.999
Non-Hispanic	18 (94.7%)	11 (91.7%)	7 (100.0%)	
Unknown ethnicity	1 (5.3%)	1 (8.3%)	0 (0.0%)	

**Table 2:** Treatment-related adverse events by study arm. Adverse events are summarized by toxicity category and graded according to the National Cancer Institute Common Terminology Criteria for Adverse Events (CTCAE), Version 5.0. Values represent the number (percentage) of participants experiencing each event.

Toxicity Category	Adverse Event	Group A + B * (Treatment Arm)			Group C (Placebo)		
		Grade 1	Grade 2	Total	Grade 1	Grade 2	Total
Gastrointestinal disorders	Flatulence	1 (8.3%)	–	1 (8.3%)	–	–	–
	Gastroesophageal reflux disease	1 (8.3%)	–	1 (8.3%)	–	–	–
	Vomiting	1 (8.3%)	–	1 (8.3%)	1 (14.3%)	–	1 (14.3%)
	Bloating	–	–	–	1 (14.3%)	–	1 (14.3%)
	Diarrhea	–	–	–	1 (14.3%)	1 (14.3%)	2 (28.6%)
	Nausea	–	–	–	1 (14.3%)	–	1 (14.3%)
Metabolism and nutrition disorders	Hypercalcemia	1 (8.3%)	–	1 (8.3%)	–	–	–
	Hyperglycemia	1 (8.3%)	–	1 (8.3%)	–	–	–
	Hyperkalemia	1 (8.3%)	–	1 (8.3%)	–	–	–
Renal and urinary disorders	Hematuria	1 (8.3%)	–	1 (8.3%)	–	–	–

* Group A + B includes participants receiving curcumin (8 g/day) plus either low-dose (2 g/day) or high-dose (4 g/day) ω-3 fatty acids. Because the low-dose group enrolled only three participants, the intervention groups were pooled for safety analyses.

**Table 3:** (a). Overall baseline-to-end-of-treatment ratio of the sum of the longest diameters of subsolid nodules. Values are presented as median (range) for all evaluable nodules. (b). Baseline-to-end-of-treatment ratio of the sum of the longest diameters of subsolid nodules by study arm. Values are presented as median (range) by study.

(**a**)			
**Overall Descriptive Statistics (Median [Range])**			
	**[ALL]**	**n**	
	n = 18		
sum_long_diam	0.85 [0.00; 1.24]	18	
(**b**)			
**Crosstabulation Arm vs. Ratios (Median [Range])**			
	**A + B (Intervention Arms)**	**C (Placebo)**	**n**
	n = 11	n = 7	
sum_long_diam	0.85 [0.00; 1.24]	0.90 [0.00; 1.04]	18

**Table 4:** Between-group comparisons of baseline-to-end-of-treatment ratios of the sum of the longest diameters of subsolid nodules. Values represent patient-level aggregated ratios (end-of-treatment divided by baseline). Between-group comparisons were performed using the Wilcoxon rank-sum test (primary analysis), with Welch *t*-test and Kruskal–Wallis test conducted as sensitivity analyses. *p* values are exploratory and not adjusted for multiple comparisons.

Test	Statistic	df	*p* Value
Welch *t*-test	−0.410	15.28	0.688
Wilcoxon rank-sum (Mann–Whitney U)	35.500	—	0.820
Kruskal–Wallis	0.075	1.00	0.785

**Table 5:** Baseline-to-end-of-treatment ratios of LDCT-derived nodule characteristics by study arm, demonstrating internal consistency across imaging endpoints. Values are presented as median (interquartile range). Ratios are defined as end-of-treatment values relative to baseline measurements. *p* values represent comparisons between study arms. LDCT-derived metrics include size-based parameters (e.g., mean diameter, volume, sum of longest diameters) and density-based parameters (e.g., average density, solid and ground-glass opacity components).

Nodule Characteristics	A + B (Intervention Arms)	C (Control)	Overall *p*
	n = 11	n = 7	
nodule_mean_diameter_AR	0.82 [0.12; 0.87]	0.86 [0.60; 1.00]	0.4653
nodule_solid_portion_mean_diam_AR	0.80 [0.08; 1.00]	0.67 [0.17; 1.00]	1.0000
number_nodules > 4 mm_AR	1.00 [0.25; 1.00]	1.00 [0.88; 1.00]	0.6248
nodule_volume_AR	0.52 [0.02; 0.77]	0.54 [0.33; 0.81]	0.7848
nodule_average_density_AR	0.91 [0.17; 1.09]	1.06 [0.73; 1.10]	0.5240
nodule_solid_portion_volume_AR	0.83 [0.01; 0.99]	0.30 [0.12; 0.93]	0.8190
nodule_percent_solid_without_air_AR	0.82 [0.11; 1.21]	1.03 [0.09; 1.45]	0.7487
nodule_percent_solid_with_air_AR	0.82 [0.11; 1.22]	1.03 [0.08; 1.46]	0.8908
nodule_solid_portion_density_AR	0.93 [0.01; 1.00]	0.31 [0.14; 1.01]	0.8902
nodule_ggo_portion_density_AR	0.89 [0.22; 1.00]	1.00 [0.68; 1.00]	0.7453
nodule_ggo_portion_density_incl_parenchyma_AR	1.00 [0.22; 1.25]	1.00 [0.68; 1.00]	0.7821
sum_of_longest_diameters_of_subsolid_nodules_AR	0.85 [0.12; 0.97]	0.90 [0.59; 0.94]	0.7848

Diam = diameter; GGO = ground-glass opacity; Dens = density; Paren = parenchyma.

## Data Availability

As required by NIH rules, we will make the data collected as part of this protocol available to outside investigators. To maintain compliance with HIPAA regulations, our preferred method will be to execute a data sharing agreement with the requestor for a limited use dataset as defined by the US Department of Health and Human Services (DHHS).

## Abbreviations

The following abbreviations are used in this manuscript:

**Abbreviation**	**Description**
5-LOX	5-Lipoxygenase
ω-3 FA	Omega-3 Fatty Acid
AE(s)	Adverse Event(s)
CBC	Complete Blood Count
CMP	Complete Metabolic Profile
COX-2	Cyclooxygenase-2
CRFs	Case Report Forms
CT	Computed Tomography Scan
CTC	Common Terminology Criteria
CTCAE	Common Terminology Criteria Adverse Events
CUR	Curcuminoids
CycD1	Cyclin-D1
DHA	Docosahexaenoic Acid
ECOG	Eastern Cooperative Oncology Group
EGFR	Epidermal Growth Factor Receptor
EPA	Eicosapentaenoic Acid
FDA	Food and Drug Administration
GI	Gastrointestinal
HU	Hounsfield Scale
IEBs	Intermediate Endpoint Biomarker(s)
IĸB	IkappaB Kinase
IĸB-α	IkappaB Kinase-Alpha
IL-1	Interleukin-1
IL-6	Interleukin-6
IL-8	Interleukin-8
IL-12	Interleukin-12
IND	Investigational New Drug Application
IRB	Institutional Review Board
LC-MS-MS	Liquid Chromatography–Tandem Mass Spectrometry
LDCT	Low-Dose Computed Tomography
LM	Lipid Mediator
LM-SPM	Lipid Mediator
LX	Lipoxine
MaR	Maresin
MaR1	Maresin 1
MCC	Moffitt Cancer Center
MCM2	Mini-Chromosome Maintenance Protein
MD(s)	Medical Doctor(s)
MMP-9	Matrix Metallopeptidase 9
NF-ĸB	Transcription Factor
NPD1/PD1	Neuroprotectin D1/Protectin D1
PA	Physical Activity
PD	Protectin
PGE2	Prostaglandin E2
PI	Principal Investigator
RvD1	Resolvin D1
RvD2	Resolvin D2
RvD3	Resolvin D3
RvE1	Resolvin E1
Rv	Resolvin
SCC	Squamous Cell Carcinoma
SF-36	Short-Form 36 Medical Outcomes Study
SPM	Specialized Lipid Mediator
SSN(s)	Subsolid Nodule(s)
Stat3	Signal Transducer and Activator of Transcription 3
TNF	Tumor Necrosis Factor
TNF-α	Tumor Necrosis Factor Alpha
QOL	Quality of Life
